# Reduced pulmonary arterial compliance predicts poor short-term outcome in children with pulmonary arterial hypertension independent of pulmonary vascular resistance

**DOI:** 10.3389/fcvm.2025.1526435

**Published:** 2025-05-22

**Authors:** Eva Gouwy, Mark-Jan Ploegstra, Meindina G. Haarman, Marcus T. R. Roofthooft, Rolf M. F. Berger, Johannes M. Douwes

**Affiliations:** Department of Pediatric Cardiology, Center for Congenital Heart Diseases, Beatrix Children’s Hospital, University Medical Center Groningen, University of Groningen, Groningen, Netherlands

**Keywords:** pulmonary vascular resistance, pulmonary arterial stiffness, resistance-compliance-time, prognosticator, survival, pulmonary arterial hypertension, children

## Abstract

**Background and study aim:**

Pulmonary arterial hypertension (PAH) is a progressive pulmonary vascular disease with pulmonary vascular remodelling leading to an increased pulmonary vascular resistance (PVRi) and decreased pulmonary arterial compliance (PACi). It is debated whether PACi provides prognostic information additional to PVRi and whether the relationship between PVRi and PACi, expressed as their product resistance-compliance-time (RC-time), is constant. The aim of this study is to investigate the relationship between PVRi and PACi and to determine the prognostic value of PACi in addition to PVRi in newly diagnosed children with idiopathic or heritable pulmonary arterial hypertension (IPAH/HPAH).

**Methods:**

In this retrospective analysis of prospectively collected data, 62 consecutive children with IPAH/HPAH that had a diagnostic heart catheterization between 1993 and 2021 at the University Medical Center Groningen were included. The relationship between PACi and PVRi was assessed using curve estimation and linear regression analysis, the relationship of PACi with disease severity was assessed using Spearman correlation and the relationship of PACi with outcome was assessed using cox regression analysis.

**Results:**

A power curve with formula PACi = 5.13 · PVRi^−0.66^ was the best fit for the relationship between PACi and PVRi, and RC-time varied between patients. PVRi and PACi both correlated with markers of disease severity and transplant-free survival. PACi was associated with 1-year transplant-free survival independent of mean pulmonary arterial pressure (mPAP) (*p* = 0.031), PVRi (*p* = 0.029), and age, sex and treatment intensity (*p* = 0.019).

**Conclusion:**

The relationship between PACi and PVRi is not constant. PACi is associated with disease severity and predicts short term outcome in children with IPAH/HPAH independent of PVRi and mPAP. Therefore, PACi is of additional prognostic value to PVRi, especially for short term prediction.

## Introduction

Pulmonary arterial hypertension (PAH) is a rare, progressive pulmonary vascular disease with characteristic pulmonary vascular remodeling causing arterial wall thickening and stiffening and occlusion of small, distal arteries (1). Consequently, right heart afterload increases, eventually resulting in right heart failure and death. PAH is diagnosed by invasive hemodynamic measurements derived during heart catheterization (HC). In children, idiopathic PAH (IPAH) and heritable PAH (HPAH) are amongst the most common forms of PAH. IPAH is a diagnosis per exclusion and HPAH is diagnosed when PAH has familial occurrence or associated gene mutations are found (2–4).

Treatment options in PAH consist of PAH-targeted drugs (including endothelin receptor antagonists, phosphodiesterase type 5 inhibitors and prostacyclin analogues) and procedural treatments to relieve the right ventricle (RV) and preserve cardiac output (atrial septostomy, Potts shunt) (3). Despite the availability of these treatment options, the prognosis of pediatric PAH remains poor, leading to death or necessity of (heart)lung transplantation. Current treatment strategies of PAH are based on risk stratification, but studies reporting risk stratification in pediatric patients are scarce, with moderate prognostic value (3, 5). Therefore, there is an urgent need for accurate prognosticators in children with PAH to improve risk stratification and treatment strategies, to achieve better outcomes (5).

In PAH, the increase of RV afterload is an important determinant of prognosis, and consists of multiple components, including indexed pulmonary vascular resistance (PVRi), indexed pulmonary arterial compliance (PACi) and so-called reflection waves. PVRi, a measure of the steady component of RV afterload, is determined by the resistance of the smallest arteries and arterioles to steady flow. PACi is a measure of the pulsatile component of RV afterload formed by the compliance of larger, elastic conduit arteries (6). In patients with PAH, remodeling of the pulmonary arterioles increases PVRi while stiffening of the conduit arteries reduces PACi, by which the latter becomes an increasingly important determinant of afterload (7). The product of PACi and PVRi has the unit of time and is referred to as resistance-compliance-time (RC-time) (8, 9).

PVRi and PACi have been shown to be predictors of outcome in both adult and pediatric PAH (4, 8, 10–13). However, the independent value of PACi as a prognosticator is controversial, since it has been debated whether or not the relationship between PACi and PVRi is constant.

The relationship between PVRi and PACi is mostly described as an inverse curvilinear relation (14–16). The relation between PVRi and PACi has been proposed to be constant, with a constant RC-time in health and disease (14, 17, 18). However, this concept of RC-time constancy has been challenged, because great RC-time variability was reported in adults with PAH (15, 16, 19, 20). If indeed there is no constant relation between PVRi and PACi, then knowledge of one (either PVRi or PACi) is not sufficient to reliably derive the other and PACi would be a prognosticator of interest in addition to PVRi.

The relation between PVRi and PACi has been insufficiently studied in pediatric PAH. We hypothesized that there is no constant relationship between PVRi and PACi in children with IPAH/HPAH. Furthermore, this study aims to determine the prognostic value of PACi in children with IPAH/HPAH in relation to PVRi.

## Materials and methods

In the Netherlands, all children with PAH are diagnosed and have standardized follow up at the Dutch national referral center for pulmonary hypertension in childhood. Patient data is prospectively collected in the national registry for pediatric pulmonary hypertension (PH) (21).

In the current longitudinal study, all children who were newly diagnosed with IPAH/HPAH by diagnostic HC between 1993 and August 2021 according to contemporary guidelines for diagnosis of PAH (mean pulmonary arterial pressure (mPAP) ≥ 25 mmHg, pulmonary arterial wedge pressure (PAWP) ≤ 15 mmHg and a pulmonary vascular resistance index (PVRi) ≥ 3 Wood units (WU) · m^2^) (22), were included from the national registry. For a proportion of the current patient population PACi has been reported previously by our group (10). Patients in whom both PVRi and PACi could not be determined were excluded from the study. Children with mPAP 21–24 mmHg who are considered to have pulmonary hypertension only according to the most recent redefined definition were not included (3). IPAH was diagnosed per exclusion of a known cause for PAH and HPAH was diagnosed when familial occurrence or PAH associated gene mutations were found (3, 4). Children with coincidental defects were not excluded because these defects do not lead to childhood PAH and are regarded IPAH (23).

Patient specific variables including date of diagnosis, age, sex and body surface area (BSA) were collected at the moment of HC. HC was performed under general anesthesia following standardized protocol including both right and left catheterization, measuring heart rate (hr), pressure, oxygen saturation and pO_2_ in systemic veins, all cardiac compartments, pulmonary artery and aorta. From the HC, mPAP, pulmonary artery diastolic pressure (PADP), systolic pressure (PASP) and PAWP were derived. Systemic and pulmonary blood flows (Qs and Qp) were calculated by Fick's method based on estimated oxygen consumption $\left(\dot{V}O_{2}\right)$. For optimal accuracy, $\dot{V}O_{2}$ was estimated using (i) formula $\dot{V}O_{2}=1.39\cdot \mathrm{height}(\mathrm{cm})+0.84\cdot \mathrm{weight}(\mathrm{kg})-35.7$ for children <7 years of age and (ii) the tables of Lafarge and Miettinen for children >7 years of age (24, 25). Pulmonary blood flow was indexed for BSA (Qpi).

Pulmonary stroke volume index (PSVi) was calculated:

$$\;\mathrm{PSVi}=\frac{\mathrm{Qpi}}{\mathrm{hr}}$$

PVRi and PACi were calculated:

$$\;\mathrm{PVRi}=\frac{(\mathrm{mPAP}-\mathrm{PAWP})\;}{\mathrm{Qpi}}$$

$$\;\mathrm{PACi}=\frac{\mathrm{PSVi}}{\left(\mathrm{PASP}-\mathrm{PADP}\right)}$$

RC-time is the product of PVRi and PACi and has the unit of min · 10^−3^, which can be transferred to seconds for comparability to previously reported studies by multiplying with 0.06.

RC-time=PVRi⋅PACi⋅0.06

Hemodynamic parameters were determined both at baseline and at acute vasodilator response testing (AVT). AVT was performed with inhaled nitric oxide (40 ppm), whether or not combined with 100% oxygen inhalation. The maximal vasodilator response was defined by the lowest PVRi reached without reduction in cardiac output (3, 26).

The best fit relationship between PVRi and PACi was determined by SPSS curve estimation function. The relationship was analyzed further by simple linear regression of the natural logarithms of PACi and PVRi. Additionally, the relationship was analyzed using spline analysis and ANOVA to compare the relationship between PVRi and PACi in AVT versus baseline conditions.

We defined disease severity by: (a) biomarkers of functional status: World Health Organization functional class (WHO-FC) and 6 minute-walking-distance (6MWD); (b) serum biomarkers: N-terminal prohormone brain natriuretic peptide (NT-pro-BNP), uric acid; and (c) echocardiographic parameters: tricuspid annular plane systolic excursion (TAPSE) and pulmonary artery acceleration time (PAAT) (4, 8). *Z*-values based on reference values (27) were used instead of absolute meters of the 6MWD to adjust for different age and corresponding height and weight of children. The correlation of PVRi, PACi and RC-time with markers of disease severity was assessed using Spearman correlation coefficient because of not normally distributed data.

Outcome analyses were based on transplant-free survival, where death or (heart)lung transplant were considered endpoints. Patients with no endpoint were censored at their last follow-up visit. The association of PVRi and PACi with 1-, 5- and 10-year transplant-free survival was analyzed by univariate cox regression analysis on data truncated at the respective time points. To further explore these associations Kaplan Meier analysis and log rank test were performed in patients with low versus high risk based on PACi. The cutoff value for PACi was determined using a ROC-curve and Youden's index and compared to the cutoff recommended by the pediatric task force of the 7th World Symposium on Pulmonary Hypertenstion (WSPH) (23). Descriptive statistics in patients with low versus high PACi were compared using Chi-square test for categorical variables and Mann Whitney U test for numerical variables. Multivariate cox regression analysis was used to correct the outcome analyses of PACi for mPAP and PVRi separately and subsequently for age, sex and treatment intensity. The correction for age, sex and treatment intensity is regarded an exploratory result because of limited numbers of events per correction factor.

Patients were treated according to contemporary treatment recommendations. The intensity of treatment regimens may vary between patients due to either changes in treatment recommendations over time or variation of disease severity between patients. Currently three groups of PAH targeted therapies (endothelin receptor antagonists, phosphodiesterase type 5 inhibitors and prostacyclin analogs) are used as mono-therapy, dual or triple combination therapy. To correct outcome analyses for the treatment patients received during the studied period, a treatment intensity score was calculated that expressed the average of the mono, dual or triple therapy a patient received during the study period. The score was calculated using the sum of time a patient used a targeted therapy, divided by the follow-up time. This results in a treatment intensity score ranging from 0 to 3, with 0 indicating no therapy and 3 indicating triple therapy during the entire follow-up period.

Statistical analyses were performed using IBM SPSS statistics 28.0 and R statistics. A 2-tailed alpha level of <0.05 was considered statistically significant.

The protocol for the Dutch national patient registry and the use of its data for observational studies was approved by the Medical Ethics Review Board of the University Medical Center Groningen (METc 2008.009). Research was conducted according to the World Medical Association Declaration of Helsinki. Written informed consent was obtained from patients and their legal guardians.

## Results

Sixty-two newly diagnosed patients (29 males, 33 females) with a median age of 7.96 were included in this study, of which 42 patients (69%) had IPAH and 19 (31%) HPAH. Thirty-nine patients (63%) were in WHO-FC III or IV. Median PVRi, mPAP and PACi were respectively 16.0 (7.8, 24.2) WU · m^2^, 51 (36, 68) mmHg and 0.9 (0.6, 1.2) ml/mmHg/m^2^ (Table 1). At the moment of HC 40 patients (65%) were treatment naïve, 9 (15%) were on PAH targeted mono-therapy, 7 (11%) on PAH targeted duo-therapy, 3 (5%) on PAH targeted triple-therapy, and 3 (5%) on calcium channel blocker mono-therapy.

**Table 1:** Patient Characteristics.

Variable	All patients (*n* = 62)	
	*n*	Value
Sex	62	
Male		29 (47)
Female		33 (53)
Age (years)	62	7.96 (2.91, 13.36)
Diagnosis	62	
IPAH		43 (69)
HPAH		19 (31)
WHO-FC	60	
I		4 (7)
II		17 (28)
III		31 (52)
IV		8 (13)
Follow-up time (years)	62	4.95 (1.41, 10.40)
mRAP (mmHg)	62	6 (4, 8)
Cardiac index (l/min/m^2^)	61	3.0 (2.4, 3.6)
mPAP/mSAP	62	0.9 (0.6, 1.2)
mPAP (mmHg)	62	51 (36, 68)
mPAP at AVT (mmHg)	59	47 (31, 60)
PVRi (WU · m^2^)	61	16.0 (7.8, 24.2)
PVRi at AVT (WU · m^2^)	59	11.7 (4.3, 20.3)
PACi (ml/mmHg/m^2^)	58	0.9 (0.6, 1.2)
PACi at AVT (ml/mmHg/m^2^)	57	1.2 (0.9, 2.0)
RC-time (s)	58	0.8 (0.6, 1.)
RC-time at AVT (s)	57	0.7 (0.5, 1.0)

Values are in median (*Q*1, *Q*3) or *n* (%) as appropriate.

IPAH, idiopathic pulmonary arterial hypertension; HPAH, heritable pulmonary arterial hypertension; WHO-FC, World Health Organization functional class; mRAP, mean right atrial pressure; mPAP, mean pulmonary arterial pressure; mSAP mean systemic arterial pressure; PVRi, pulmonary vascular resistance indexed for body surface area; PACi, pulmonary arterial compliance indexed for body surface area; RC, resistance-compliance; AVT, acute vasodilator response testing; s, seconds; min, minute.

**Figure 1:**
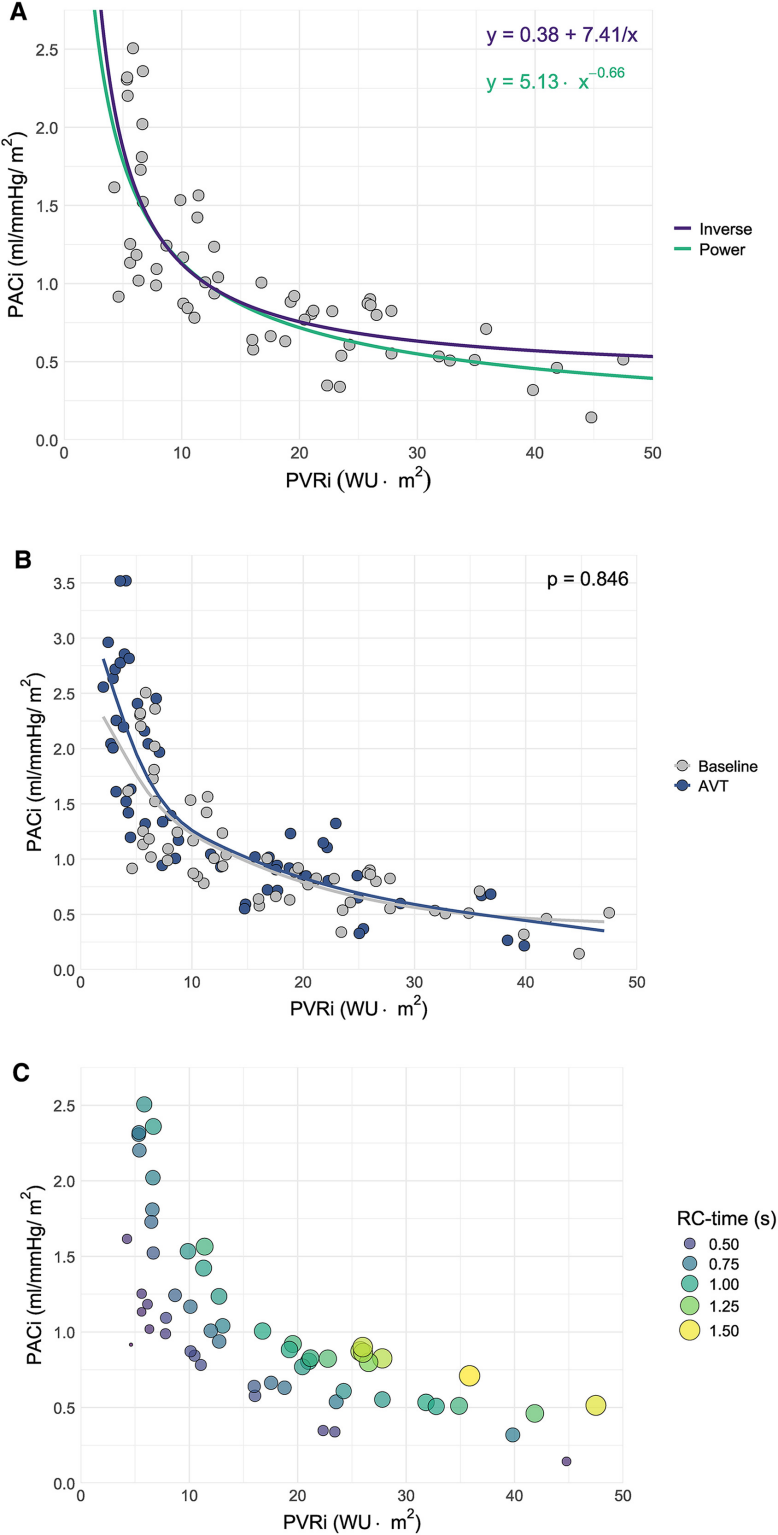
Scatter plot of pulmonary arterial compliance (PACi) versus pulmonary vascular resistance (PVRi) including **(A)** The curves corresponding to the power and inverse relationships between PACi and PVRi derived from curve estimation analysis, **(B)** Spline estimation of the PACi-PVRi relationship at baseline versus acute vasodilator response testing (AVT) and **(C)** resistance-compliance-time (RC-time) in seconds (s) as visualized by the size and color of the dots.

A power curve with formula $y=5.13\cdot x^{-0.66}$ was demonstrated to be the best fit for the relationship between PVRi and PACi in this patient group (R^2^ 0.65, *p* < 0.001). Although an inverse curve with formula y=0.38+(7.41/x) was also significant, this relation appeared less strong (R^2^ 0.60, *p* < 0.001) (Figure 1A).

There was a significant negative linear relationship between the natural logarithm of PACi and PVRi at baseline measurement with beta −0.657 (−0.786 to −0.528) *p* < 0.001 and at AVT with beta −0.640 (−0.745 to −0.535) *p* < 0.001. There was no significant difference in the relationship between PACi and PVRi at baseline and AVT (*p* = 0.846) (Figure 1B).

**Figure 2:**
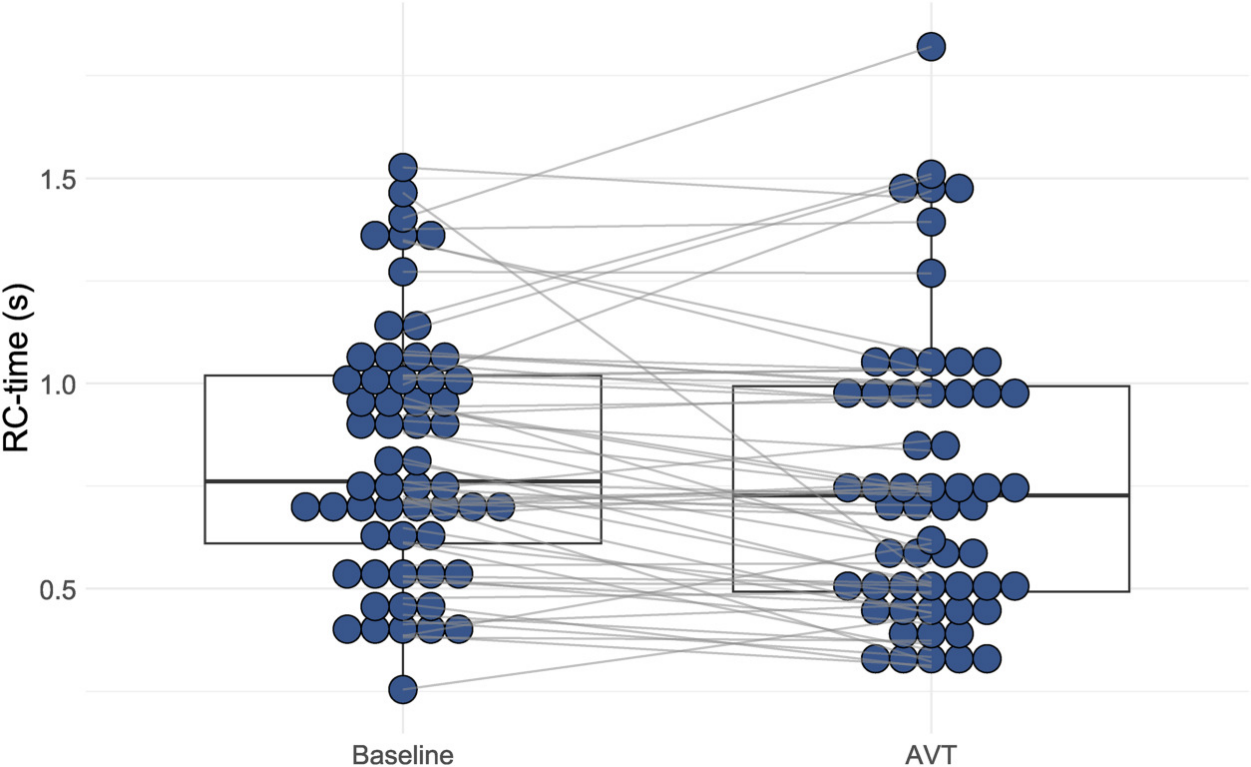
Distribution of resistance-compliance-time (RC-time) during baseline conditions and acute vasodilator testing (AVT). There is a statistically significant difference between RC-time at baseline conditions versus at AVT (Wilcoxon Signed Rank test *p* < 0.001).

Constancy of the PACi and PVRi relationship would mean a constant RC-time. However, RC-time had a large (>30%) coefficient of variation (38% at baseline and 46% at AVT) caused by a large standard deviation of RC-time. This large variation is illustrated by the variation of RC time for comparable PVRi values (Figure 1C, RC-time represented in the size and color of the dots). Furthermore, the RC-time at AVT was significantly different from the RC-time at baseline (*p* < 0.01) (Figure 2).

Both lower PACi and higher PVRi correlated significantly with worse WHO-FC, higher NT-pro-BNP, higher uric acid and lower PAAT. A higher RC-time was associated with a higher uric acid and lower PAAT (Table 2). No significant associations of PVRi, PACi and RC-time with TAPSE and 6MWD could be demonstrated.

**Table 2:** Spearman correlation of PACi, PVRi and RC-time with markers of disease severity.

Variable	Markers of disease severity			
	WHO-FC	NT-pro-BNP	Uric acid	PAAT
PACi	−0.447 *p* < 0.001	−0.519 *p* < 0.001	−0.405 *p* = 0.006	0.521 *p* < 0.001
PVRi	0.465 * p* < 0.001	0.430 *p* = 0.004	0.522 *p* < 0.001	−0.588 *p* < 0.001
RC-time			0.312 *p* = 0.039	−0.469 *p* = 0.003

PACi, pulmonary arterial compliance index; PVRi, pulmonary vascular resistance index; RC, resistance-compliance; WHO-FC, World Health Organization functional class; NT-pro-BNP, N-terminal prohormone brain natriuretic peptide; PAAT, pulmonary arterial acceleration time.

**Figure 3:**
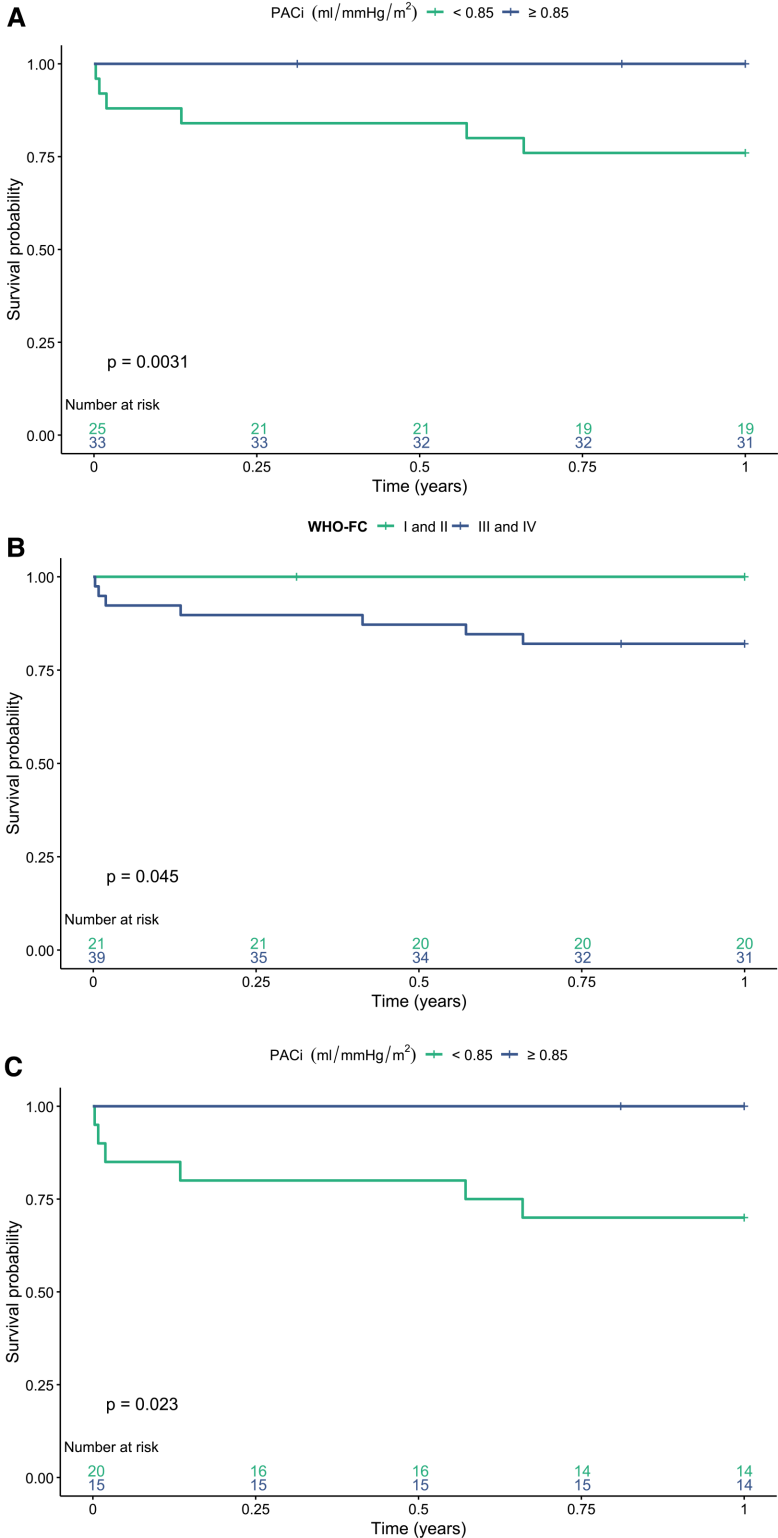
One year transplant-free survival stratified for **(A)** low versus high pulmonary arterial compliance index (PACi) in the whole study group. **(B)** World Health Organization functional class (WHO-FC) and **(C)** WHO-FC and PACi. There is a significant difference in transplant-free survival between patients with a high versus low PACi [**(A)** log rank test *p* = 0.003] and WHO-FC I or II versus WHO-FC III and IV [**(B)** log rank test *p* = 0.045]. There is a significant difference in 1-year transplant-free survival between a PACi <0.85 versus ≥0.85 ml/mmHg/m^2^ in patients in WHO-FC III or IV [**(C)** log rank test *p* = 0.023].

The 1-, 5- and 10-year transplant-free survival of the study cohort was 89%, 66% and 45%, respectively. PACi was associated with 1-year transplant-free survival independent of mPAP [0.66 (0.46–0.96) *p* = 0.031] and PVRi [0.58 (0.35–0.95) *p* = 0.029] (hazard ratio per 0.1 ml/mmHg change in PACi). Furthermore, PACi was associated with 1- and 5-year transplant-free survival independent of age, sex and treatment intensity. PVRi was associated with 1-, 5-, and 10-year transplant-free survival independent of age, sex and treatment intensity (Table 3). The ROC-curve analysis revealed a PACi of 0.85 ml/mmHg/m^2^ to discriminate high risk (*n* = 25) from low risk (*n* = 33) patients for 1 year transplant-free survival (Figure 3A, *p* = 0.003). Groups based on low versus high PACi were similar in regard to age, sex, diagnosis and follow-up time. WHO-FC and hemodynamics including RC-time at AVT differed significantly between the groups (Supplementary Table S1). Given the correlation between PACi and the non-invasive predictor of outcome WHO-FC, we investigated whether PACi had additional predictive value to WHO-FC for 1-year survival. Patients with WHO-FC III or IV had worse outcome compared to patients in WHO-FC I or II (Figure 3B, *p* = 0.045). Within the patients with WHO-FC III or IV, patients with higher PACi (cut off at 0.85 ml/mmHg/m^2^) had better survival (Figure 3C, *p* = 0.023). The WSPH cutoff for PACi of 0.9 ml/mmHg/m^2^ also discriminated well between low risk versus high risk patients in the whole patient group (Supplementary Figure S1A, log-rank test *p* = 0.011). However, PACi cutoff value 0.9 ml/mmHg/m^2^ discriminated less well for transplant-free survival in patients with WHO-FC III and IV (Supplementary Figure S1B, log-rank test *p* = 0.06).

**Table 3:** Outcome analysis for transplant-free survival.

Time of data truncation	Variable	Median for Event:		Cox regression analyses			
				Univariate	Corrected for mPAP	Corrected for PVRi or PACi^a^	Corrected for age, sex and treatment intensity
		Yes	No				
**1 year**	PVRi	23.1	12.8	1.06 (0.99–1.13)		0.95 (0.85–1.06)	1.09 (1.02–1.17)**
7 events	PACi	0.5	0.9	0.70 (0.52–0.93)* ^b^	0.66 (0.46–0.96)* ^b^	0.58 (0.35–0.95)* ^b^	0.65 (0.45–0.93)* ^b^
**5 year**	PVRi	22.4	11.7	1.04 (1.00–1.07)*		1.0 (0.94–1.06)	1.05 (1.01–1.09)*
20 events	PACi	0.8	1.0	0.86 (0.75–0.98)* ^b^	0.89 (0.75–1.05)^b^	0.85 (0.69–1.05)^b^	0.84 (0.72–0.98)* ^b^
**10 year**	PVRi	21.0	11.7	1.03 (1.00–1.06)*		1.03 (0.98–1.08)	1.04 (1.01–1.08)*
28 events	PACi	0.8	1.0	0.94 (0.86–1.02)^b^	0.98 (0.88–1.09)^b^	0.99 (0.88–1.12)^b^	0.93 (0.85–1.02)^b^

^a^ PACi is corrected for PVRi and PVRi is corrected for PACi.

^b^ Hazard ratio per 0.1 ml/mmHg change in PACi.

* *p* < 0.05.

** *p* < 0.01.

*** *p* < 0.001.

Univariate and multivariate cox regression analyses for 1-, 5- and 10-year transplant free survival.

PACi, pulmonary arterial compliance index; PVRi, pulmonary vascular resistance index; mPAP, mean pulmonary arterial pressure.

## Discussion

In this study of a national cohort of children with IPAH/HPAH we demonstrated a significant relationship between PACi and PVRi. However, the data showed significant variation within this statistical relationship, and consequently a large variation of RC-time. These findings confirm that RC-time is not constant in pediatric IPAH/HPAH patients. Moreover, both PACi and PVRi were significantly associated with other markers of disease severity and transplant-free survival.

Previously, in adult PAH patients, the PACi-PVRi relation has been reported to be a constant, inverse curvilinear relationship, resulting in a constant RC-time in both health and disease and thus irrespective of PAH disease progression (14, 17, 28). However, this constancy of RC-time has been challenged. Several reports have demonstrated significant RC-time variations in different hemodynamic conditions and within PH disease subgroups (15, 16, 18–20). These observations challenge the concept of constancy in the PACi-PVRi relationship. Whether the PACi-PVRi relationship behaves similar in children is not known. As far as we are aware there are no studies directly comparing the correlation of PACi and PVRi in children to the correlation in adults.

Is has been suggested that in adults PACi and RC-time vary with the etiology of PH (19). Since etiology of PH differs markedly between children and adults, one may speculate that also the PACi-PVRi relation does. Furthermore, PAH in childhood is frequently associated with injury of the developing pulmonary vasculature, leading to disturbed pulmonary vascular and parenchymal development. This disturbed vascular development may affect pulmonary arterial stiffness and hemodynamics and thus PACi-PVRi relation (23, 29, 30). Additional comparative research is essential to clarify these potential differences.

Based on the concept of a constant PACi-PVRi relationship it has been postulated that PACi would not have any prognostic or clinical value in PAH patients in addition to PVRi. Here we need to make the distinction between a statistical relationship and the concept of constancy. We found on average a statistically significant relationship between PACi and PVRi. However PVRi and PACi measurements of individual patients vary around this relationship. As RC-time is not constant a certain PVRi may be accompanied by either a high or low PACi. Since PACi decreases in patients with PAH and is an important determinant of RV afterload, a low PACi may have different prognostic implications in comparison to higher PACi, even if accompanied by comparable PVRi.

In our current study we found that PACi predicted 1-year transplant-free survival independent of PVRi, mPAP and treatment intensity. The prognostic value of PACi has been previously reported in adult and pediatric PAH (4, 10–13). We now add to these reports that in pediatric IPAH/HPAH PACi is especially valuable as an independent predictor of short-term outcome including transplant-free survival. Given the low PACi in the patients that die or undergo lung transplantation within 1 year of follow-up it is apparent that especially patients with a low PACi are at risk of short-term adverse outcome. PVRi was also shown a predictor of outcome in our cohort of pediatric IPAH/HPAH patients, but not an independent predictor for the 1-year outcomes. PACi adds a strong prediction of short term outcome, independent of and as such complementary to PVRi.

Also, PACi seems to have additional prognostic value to the non-invasive parameter WHO-FC. In children who are predicted to have a worse prognosis based on their WHO-FC, PACi seems to be able to further identify the patients that are especially at high risk of short term death. In our patient cohort, we found a PACi cutoff value of 0.85 ml/mmHg/m^2^ to be most predictive of 1-year transplant-free survival. Although the WSPH cutoff value of 0.9 ml/mmHg/m^2^ also showed to have predictive value in the whole study population, especially for patients in WHO-FC III and IV, 0.85 ml/mmHg/m^2^ seemed to discriminate better between high and low risk by correctly classifying 3 more patients as low risk. The threshold of 0.85 ml/mmHg/m^2^ has been previously reported in a cohort with Japanese children with PAH by Takatsuki et al. (12). The current refinement of the PACi cutoff value for children with PAH follows the call of the WSPH pediatric task force to validate the proposed cutoff values for risk factors in independent cohorts. These validation studies in different cohorts are of great importance for the clinical use of risk factors for risk stratification in children with PAH. The current study provides an addition to and extension of our groups publication of 2013 (10) by providing additional insights into the relationship between PACi and PVRi and the lack of RC-time constancy in a pediatric cohort. Also, this study shows the additional prognostic value of PACi on specifically short term survival in a less heterogenous group with exclusively IPAH/HPAH patients.

The short-term prognostic value of PACi, suggests that pulmonary arterial stiffening and reduced PACi occurs in more advanced and severe PAH. This results may seem contradictory to research that states that change of PACi is an early phenomenon in disease course (14, 31). Based on the PVRi-PACi relationship the PACi is expected to decrease significantly early in the disease course, while PVRi is not yet very much increased. That first rapid decrease of PACi may be the consequence of increase in mPAP leading to a circumferential stretch of the pulmonary arterial wall, resulting in a decrease of PACi due to the viscoelastic properties of the pulmonary arterial wall (32). The short-term prognostic ability of PACi was independent of PVRi and mPAP and as such may reflect intrinsic pulmonary arterial wall stiffening, due to advanced pulmonary vessel wall remodeling. The natural course of pulmonary arterial stiffening in PAH however has yet been insufficiently studied, prohibiting definite conclusions on this aspect.

Limitations of our study lie in the inevitable limitations of a retrospective analysis of prospectively collected patient data in pediatric patients with a rare disease. A low number of patients and heterogeneity are inherent to the pediatric PAH population, however in this study heterogeneity was limited by including exclusively IPAH/HPAH patients. The results may not be extrapolated to other types of PAH. More data points in the higher PVRi and PACi regions would have allowed for further enhancement of accuracy in characterization of the relationship between PACi and PVRi. In this study $\dot{V}O_{2}$ was assumed and not measured, reflecting real-life clinical practice which will therefore have little effect on the clinical relevance of the study.

In contrast to the available risk stratification tools for adults with PH, there are currently no risk stratification models validated for children (5). Although some combinations of risk factors have been proposed to stratify risk in children with PAH, none of these have been validated (33–36). Recently, a risk table with individual validated risk factors has been proposed for children with PAH (23). However, these individual variables have not been studied in combination, causing the weight of each variable and their interdependency to remain unknown. Therefore, these variables cannot be used as integrated risk scores yet. The current study shows that PVRi and PACi are independent prognosticators and could be of value for multi-parameter risk models in pediatric PAH. Future research should explore the potential role of PACi and PVRi in these risk stratification models.

In conclusion, **t**he relationship of PACi with PVRi and their product RC-time were not constant in this cohort of pediatric IPAH/HPAH patients. We found PACi to be associated with markers of disease severity and predictive of short-term outcome in children with IPAH/HPAH. This predictive value of PACi for short-term outcome was additional to and independent of non-invasive parameters WHO-FC and treatment intensity as well as invasive parameters mPAP and PVRi. If validated in an independent cohort PACi may be used to improve the accuracy of current risk score tools in pediatric PAH.

## Data Availability

The datasets presented in this article are not publicly available due to patient privacy. Requests to access the datasets should be directed to E. Gouwy, e.gouwy@umcg.nl.
