# Supplementary material for: Reduced pulmonary arterial compliance predicts poor short-term outcome in children with pulmonary arterial hypertension independent of pulmonary vascular resistance
Source: Front Cardiovasc Med. 2025 May 22;12:1526435. doi: 10.3389/fcvm.2025.1526435 (PMC12139025; doi:10.3389/fcvm.2025.1526435)
Supplement: Supplementary file 1 [file Datasheet1.pdf]

Reduced pulmonary arterial compliance predicts poor short-term outcome in children with pulmonary arterial hypertension independent of pulmonary vascular resistance

Eva Gouwy*, Mark-Jan Ploegstra, Meindina G. Haarman, Marcus T. R. Roofthooft, Rolf M. F. Berger and Johannes M. Douwes

Supplementary files

Supplementary tables

Table S1 descriptive statistics in groups based on high versus low pulmonary arterial compliance index

	PACi < 0.85 ml/mmHg/m² N = 25	***PACi ≥ 0.85 ml/mmHg/m²*** N = 33	**p-value**
Sex			0.85
Male	12 (48%)	15 (45%)	
Female	13 (52%)	18 (55%)	
Age (years)	7.9 (2.3, 13.2)	9.8 (4.6, 13.8)	0.42
Diagnosis			0.22
HPAH	6 (24%)	13 (39%)	
IPAH	19 (76%)	20 (61%)	
WHO-FC			0.049
I	1 (4.2%)	3 (9.4%)	
II	3 (13%)	14 (44%)	
III	16 (67%)	12 (38%)	
IV	4 (17%)	3 (9.4%)	
Follow-up time (years)	3 (1, 9)	5 (3, 12)	0.23
mRAP (mm Hg)	6.0 (5.0, 9.0)	5.0 (4.0, 8.0)	0.31
mPAP (mm Hg)	66 (55, 79)	37 (32, 50)	<0.001
mPAP at AVT (mmHg)	59 (51, 76)	32 (25, 46)	<0.001
PVRi (WU*m^2)	24 (20, 33)	8 (6, 13)	<0.001
PVRi at AVT (WU*m^2)	18 (16, 25)	4 (4, 7)	<0.001
PACi (ml/mmHg/m^2)	0.61 (0.51, 0.78)	1.18 (0.99, 1.62)	<0.001
PACi at AVT (ml/mmHg/m^2)	0.79 (0.59, 1.01)	2.01 (1.32, 2.56)	<0.001
RC-time (s)	0.92 (0.61, 1.07)	0.72 (0.53, 0.95)	0.14
RC-time at AVT (s)	0.86 (0.54, 1.15)	0.68 (0.43, 0.84)	0.018

Values are in median (Q1, Q3) or n (%) as appropriate. All patients with pulmonary arterial compliance index (PACi) measurements (n=58) were divided into patients with high risk (PACi <0.85 ml/mmHg/m^2) and low risk (PACi ≥ 0.85 ml/mmHg/m^2). Differences in baseline characteristics were assessed using Chi-square or Mann Whitney U tests as appropriate.

PACi, pulmonary arterial compliance indexed for body surface area; IPAH, idiopathic pulmonary arterial hypertension; HPAH, heritable pulmonary arterial hypertension; WHO-FC, World Health Organization Functional Class; mRAP, mean right atrial pressure; mPAP, mean pulmonary arterial pressure; mSAP mean systemic arterial pressure; PVRi, pulmonary vascular resistance indexed for body surface area; RC, Resistance-Compliance; AVT, acute vasodilator response testing; s, seconds; min, minute.

Supplementary figures

Figure S1A

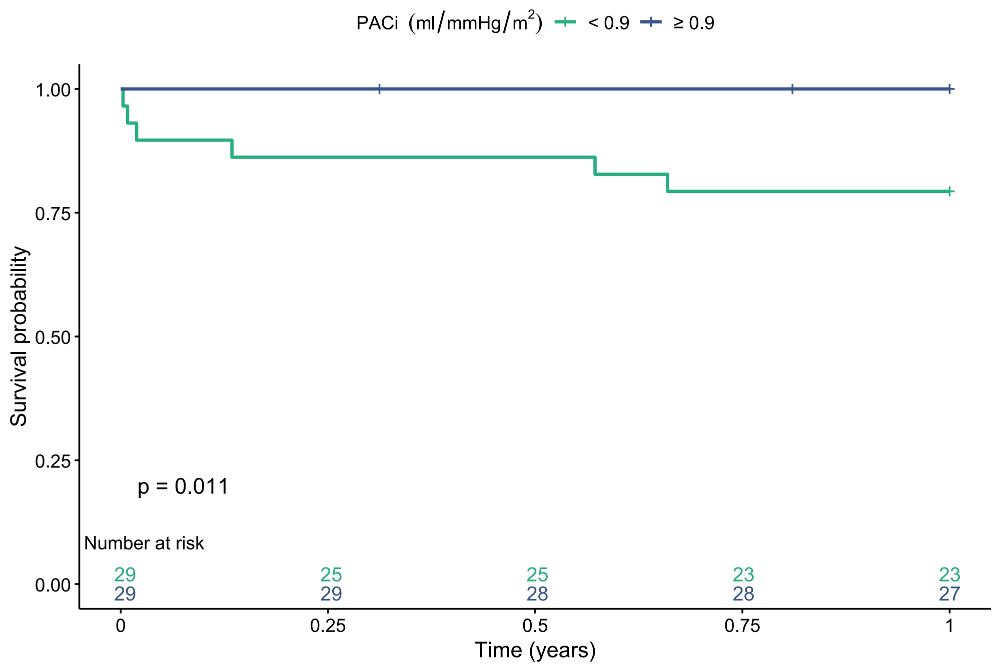

Figure S1B

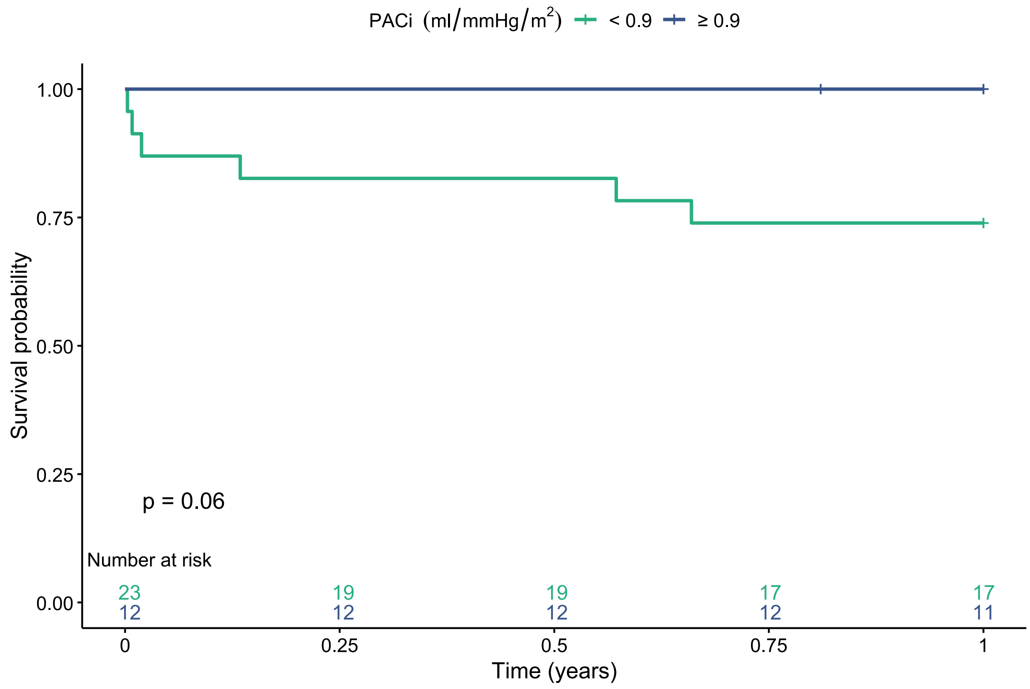

Figure S1 One year transplant-free survival stratified for low versus high risk pulmonary arterial compliance index (PACi) in subgroups based on cutoff 0.9 ml/mm Hg/m^2 as proposed by the pediatric task force of the 7th World Symposium on Pulmonary Hypertension in A) the whole study population and B) patients with World Health Organization Functional Class (WHO-FC) III and IV. One year transplant-free survival in patients with low versus high PACi significantly differs in the whole study population (A: log rank test p=0.011) but not in the patients with WHO-FC III and IV (B: log-rank test p=0.06).
